# Protocol: Evaluating the impact of a nation-wide train-the-trainer educational initiative to enhance the quality of palliative care for children with cancer

**DOI:** 10.1186/s12904-016-0085-8

**Published:** 2016-01-27

**Authors:** Kimberley Widger, Stefan Friedrichsdorf, Joanne Wolfe, Stephen Liben, Jason D. Pole, Eric Bouffet, Mark Greenberg, Amna Husain, Harold Siden, James A. Whitlock, Adam Rapoport

**Affiliations:** Lawrence S. Bloomberg Faculty of Nursing, University of Toronto, 130-155 College Street, Toronto, ON M5T 1P8 Canada; Pediatric Advanced Care Team, Hospital for Sick Children, 555 University Avenue, Toronto, ON M5G 1X8 Canada; Department of Pain Medicine, Palliative Care & Integrative Medicine, Children’s Hospitals and Clinics of Minnesota, 2525 Chicago Avenue South, Minneapolis, MN 55404 USA; Pediatric Palliative Care Service, Department of Psychosocial Oncology and Palliative Care, Dana-Farber Cancer Institute, 450 Brookline Avenue, Boston, MA 02215 USA; Pediatric Palliative Care Program, The Montreal Children’s Hospital, 2300 Rue Tupper, Montréal, QC H3H 1P3 Canada; Pediatric Oncology Group of Ontario, 480 University Avenue, Suite 1014, Toronto, ON M5G 1 V2 Canada; Brain Tumor Program, Division of Hematology/Oncology, Hospital for Sick Children, 555 University Avenue, Toronto, ON M5G 1X8 Canada; Policy and Clinical Affairs, Pediatric Oncology Group of Ontario, 480 University Avenue, Suite 1014, Toronto, ON M5G 1 V2 Canada; Department of Family and Community Medicine, University of Toronto, Temmy Latner Centre for Palliative Care, Mount Sinai Hospital, 60 Murray Street, 4th Floor, Toronto, ON M5T 3 L9 Canada; Canuck Place Children’s Hospice, Clinical Professor, Department of Pediatrics, University of British Columbia, 1690 Matthews Avenue, Vancouver, BC V6J 2 T2 Canada; Department of Hematology/Oncology, Hospital for Sick Children, 555 University Avenue, Toronto, ON M5G 1X8 Canada; Pediatric Advanced Care Team, Hospital for Sick Children, 555 University Avenue, Toronto, ON M5G 1X8 Canada

**Keywords:** Pediatrics, Palliative care, Cancer, Quality care, Education, Knowledge translation

## Abstract

**Background:**

There are identified gaps in the care provided to children with cancer based on the self-identified lack of education for health care professionals in pediatric palliative care and in the perceptions of bereaved parents who describe suboptimal care. In order to address these gaps, we will implement and evaluate a national roll-out of *Education in Palliative and End-of-Life Care for Pediatrics* (EPEC®-Pediatrics), using a ‘Train-the-Trainer’ model.

**Methods/design:**

In this study we are using a pre- post-test design and an integrated knowledge translation approach to assess the impact of the educational roll-out in four areas: 1) self-assessed knowledge of health professionals; 2) knowledge dissemination outcomes; 3) practice change outcomes; and 4) quality of palliative care. The quality of palliative care will be assessed using data from three sources: a) parent and child surveys about symptoms, quality of life and care provided; b) health record reviews of deceased patients; and c) bereaved parent surveys about end-of-life and bereavement care. After being trained in EPEC®-Pediatrics, ‘Master Facilitators’ will train ‘Regional Teams’ affiliated with 16 pediatric oncology programs in Canada. Each team will consist of three to five health professionals representing oncology, palliative care, and the community. Each team member will complete online modules and attend one of two face-to-face conferences, where they will receive training and materials to teach the EPEC®-Pediatrics curriculum to ‘End-Users’ in their region. Regional Teams will also choose a *Tailored Implementation of Practice Standards* (TIPS) Kit to guide implementation of a quality improvement project in their region; support will be provided via quarterly meetings with Co-Leads and via a listserv and webinars with other teams.

**Discussion:**

Through this study we aim to raise the level of pediatric palliative care education amongst health care professionals in Canada. Our study will be a significant step forward in evaluation of the impact of EPEC®-Pediatrics both on dissemination outcomes and on care quality at a national level. Based on the anticipated success of our project we hope to expand the EPEC®-Pediatrics roll-out to health professionals who care for children with non-oncological life-threatening conditions.

**Electronic supplementary material:**

The online version of this article (doi:10.1186/s12904-016-0085-8) contains supplementary material, which is available to authorized users.

## Background

Despite significant improvements in treatment, childhood cancer remains a leading cause of non-accidental death in children beyond infancy [1]. Annually in Canada, about 10,000 children are living with cancer (both those receiving cancer treatments and long-term survivors) and approximately 210 will die from their disease [2]. Even when a child survives, from the moment of diagnosis the threat of death may be foremost in the mind of the parents, siblings, and ill child. The treatments aimed at achieving survival are generally intensive with a significant symptom burden that is often long-lasting and disruptive to the life of the child and family [3].

Palliative care for children focuses on alleviating the physical, social, psychological, and spiritual suffering experienced by children and families, while promoting quality of life, fostering family connections, and sustaining hope despite the possibility of death. It is a family-centered approach that includes shared decision-making and sensitivity to the family’s cultural and spiritual values, beliefs, and practices [4]. In relation to pediatric cancer, palliative care has historically been thought of as being relevant only once all treatments with curative intent have been discontinued, and is offered as an alternative rather than a concurrent treatment [5]. However, emerging data suggest that principles of palliative care can and should be incorporated from diagnosis and throughout the disease course, not only at the end of life, to ensure relief of suffering and good quality of life regardless of the disease outcome [6–10]. Consultation from specialist palliative care teams may be helpful for more complex situations [3].

Oncologists and other health professionals report receiving little training specific to pediatric palliative care (PPC) [11–13]. This lack of training may contribute to reports of less than optimal care in this area: parents’ report that children with cancer experience a great deal of suffering from pain and other symptoms left inadequately treated [14, 15]; some parents feel abandoned by health professionals both before and after their child’s death [16, 17]; family-centered care is not uniformly practiced [12]; and siblings’ needs are not always adequately addressed [12, 18]. To realize improvements in these areas, it is crucial that all health professionals who provide care to children with cancer receive comprehensive education about PPC as well as guidance and support to implement new knowledge and skills throughout the disease course. Moreover, efforts are needed to enhance collaboration between pediatric oncology and specialists in PPC. Such efforts will build familiarity, trust, and relationships, thereby facilitating concurrent delivery of disease-directed and palliative care to children living with cancer [3]. To achieve these goals, we will implement a national roll-out of *Education in Palliative and End-of-Life Care for Pediatrics* (EPEC®-Pediatrics) [19], a curriculum specifically designed for pediatric physicians and advanced practice nurses using a ‘Train-the-Trainer’ model.

Built on the demonstrated success of the original EPEC™ curriculum [20], focused on care of adults, EPEC®-Pediatrics combines didactic sessions, video presentations, interactive discussions, and practice exercises. It is comprised of 24 modules that can be taught face-to-face to interprofessional End-Users by EPEC®-Pediatrics Trainers. The Trainers learn the content via 19 online modules plus 5 delivered at a one and a half day in-person conference by EPEC®-Pediatrics Master Facilitators demonstrating effective adult teaching. Funded by a US$ 1.6 million National Institutes of Health/National Cancer Institute (1 R25 CA151000-01) grant from 2010-2015, Friedrichsdorf et al. developed the curriculum in collaboration with 35 national and international leaders in the field of paediatric pain medicine, hematology/oncology and palliative care as well as parent advisors [21]. A list of the modules is provided in Table 1. Following initial development, the curriculum was refined between 2012 and 2014 based on a beta testing conference and three subsequent Train-the-Trainer conferences with 200 pediatric physicians and advanced practice nurses in the United States and 19 other countries. All EPEC®-Pediatrics content is evidence-based and is delivered according to effective adult-education pedagogy. EPEC®-Pediatrics uses a Train-the-Trainer model; participants learn the content as well as obtain effective strategies and resources to educate others. In a recent systematic review, use of a Train-the-Trainer model was shown to be effective in improving knowledge and skills of health professionals, enhancing dissemination, and having a positive impact on patient outcomes [22].

**Table 1:** EPEC®-Pediatrics modules

	Title	Mode of delivery^a^
1	Pediatric Palliative Care: Why Does it Matter	Online
2	Child Development	Online
3	Family Centered Care	Online
4	Grief and Bereavement	Online
5	Self-Care for Professionals	Face-to-Face
6	Team Collaboration and Effectiveness	Face-to-Face
7	Communication and Planning	Face-to-Face
8	Ethical and Legal Considerations	Online
9	Teaching with EPEC®-Pediatrics in the Face-to-Face Setting	Face-to-Face
10	Multimodal Analgesia	Online
11	Opioid Selection and Rotation	Online
12	Management of Neuropathic Pain and Adjuvant Analgesia	Online
13	Procedural Pain Management Strategies	Online
14	Chronic Complex Pain Management	Online
15	Management of Gastrointestinal Symptoms	Online
16	Management of Respiratory Symptoms	Online
17	Management of Emotional and Behavioral Symptoms	Online
18	Management of Neurologic Symptom	Online
19	Palliative Sedation	Online
20	Preparing for Imminent Death	Online
21	Integrative Medicine	Online
22	Introducing Quality Improvement to PPC	Online
23	Teaching Pain and Symptom Management	Face-to-Face
24	Methadone	Online

^a^Online for education of EPEC-Pediatric Trainers; all modules will be taught face-to-face to the End-User

While data on the effective dissemination of EPEC®-Pediatrics and EPEC™ is favorable [20], the impact on patient/family outcomes has not yet been measured. Recognition that education does not necessarily lead to significant improvements has led to a strong emphasis within the EPEC®-Pediatrics curriculum in each module to address “attitudes” (myths, misconceptions, obstacles) as well as providing a “skill” in addition to sharing “knowledge” in hopes of behavior changes among the Trainers and End-Users [23, 24]. To increase the chance of practice change toward better clinical pediatric care, the curriculum added the *Tailored Implementation of Practice Standards* (TIPS) Kit to the suite of resources available through EPEC™. Each TIPS Kit includes a protocol for an evidence-based clinical intervention (e.g. symptom management protocol), a guide for how to adapt the protocol to meet local needs, a template for evaluation measures, and detailed plans for quality improvement (QI) methods for implementation. TIPS Kits reduce the initial time investment needed to develop a QI project by providing a limited number of possible actions and measurement options to select from. The EPEC®-Pediatrics curriculum currently includes one TIPS Kit, which aims to standardize symptom assessment for children with serious illness using two pediatric versions of the Memorial Symptom Assessment Scale [25, 26].

In our study, the content of EPEC®-Pediatrics will not be changed; however, four key features will be added to the delivery process based on implementation science [27] to promote sustained improvement in the quality of palliative care for children with cancer and their families. 1) Rather than attending as individuals, Regional Teams of three to five health professionals representing oncology, palliative care, and the community (e.g., home care nurses, community pediatricians) will attend a Train-the-Trainer Conference together in 2015 and work as a team. 2) A second day will be added to the Conference to allow Regional Teams to review data collected in the pre-test period and use those data to develop regional dissemination strategies and plan their QI project. 3) The Research Team developed two additional TIPS Kits to provide a wider choice of QI projects. 4) Ongoing support will be provided by the Research Team for education and QI initiatives throughout the intervention period, 2015 - 2016. Teams will also communicate with each other via a listserv and webinars to share successes and challenges.

The effectiveness of our EPEC®-Pediatrics roll-out will be assessed according to four outcomes. The guiding research questions are: What is the impact of EPEC®-Pediatrics on: 1) the self-assessed knowledge of health professionals who take part in the curriculum; 2) knowledge transfer and dissemination outcomes; 3) practice change outcomes, and 4) the quality of palliative care provided to children with cancer and their families?

## Methods/design

### Design

In this study we will use a pre-post-test design and an integrated knowledge translation (KT) approach with involvement of knowledge users and key-stakeholders through the project [28]. The pre-test period will begin in January 2015 and the post-test period will begin in the fall of 2016. This timeline allows for the educational rollout of EPEC®-Pediatrics and QI to occur over approximately 15 months. Data collection and analysis will occur in three streams in order to answer the research questions: 1) knowledge dissemination, 2) quality improvement, and 3) care quality. The study has been approved by the Research Ethics Board (REB) at the Hospital for Sick Children (#1000047116) as the primary site as well as by the relevant REB for each participating site (See Additional file 1 for a list of sites with the corresponding REB).

### Stream 1 - Knowledge dissemination

Data collection and analysis in Stream 1 will address the impact of EPEC®-Pediatrics on the self-assessed knowledge of health professionals who take part in the curriculum and on knowledge transfer and dissemination outcomes.

#### Sample and procedure

The EPEC®-Pediatrics roll-out will include training of five Master Facilitators within Canada; Master Facilitators are individuals qualified to train future EPEC®-Pediatrics Trainers at the face-to-face conference. There are 3 steps to becoming a Master Facilitator: 1) become an EPEC®-Pediatrics Trainer by completing all 19 online modules and attending a face-to-face conference as a participant; 2) attend a Professional Development Workshop offered by EPEC for additional development of teaching skills, and finally 3) teach at an EPEC®-Pediatrics Train-the-Trainer conference where feedback is provided on teaching skills by existing Master Facilitators. At each of the 16 participating sites, 3 to 5 health professionals will be identified as Regional Team members. Each Regional Team member (*n* = 45-80) will become an EPEC®-Pediatrics Trainer through completion of 19 online-modules and 5 face-to-face modules. Two face-to-face sessions will be held over two full days with the content delivered by the new Canadian Master Facilitators in conjunction with the original developers of EPEC®-Pediatrics. Regional Teams will complete the online modules on their own, then attend one of the two-day sessions as a team. At the end of the face-to-face session the new Trainers will be asked to provide feedback on EPEC®-Pediatrics as a whole and assess their learning as part of the process. Feedback will be sought using a Knowledge Transfer and Exchange (KTE) Survey completed in paper based format.

Once Regional Team members have completed their training they will work to deliver aspects of the EPEC®-Pediatrics curriculum to End-Users at their local site. Trainers will be encouraged to think very broadly about who provides care to children with cancer within the institution where they are based, within the local community, and more broadly in the wider geographic area served by the hospital. Education sessions may be offered as part of existing structures (e.g., resident training session, education days for nurses, academic rounds, lunch and learns) or as separate sessions delivered specifically for the study. When End-Users attend an EPEC®-Pediatrics session, they will be provided with a consent form that gives details about our study. If they agree to take part, End-Users will be asked to complete the KTE Survey in hard copy to assess the impact of the session on knowledge transfer. Even if people in attendance do not want to take part in the research aspect of the session they are still welcome to attend. The Trainers who deliver the session will collect the surveys and submit them to the core study team via Research Electronic Data Capture (REDCap™) database, which is a secure, web-based application designed specifically for clinical research [29].

Regional Team members will be asked to complete an Education Roll-out Report on a quarterly basis to provide summary information about the education sessions they have delivered to local End-Users. The Report will be completed verbally with the Project Manager/Principal Investigators (PI) via telephone or Skype and data will be entered into REDCap™.

#### Data collection tools

The KTE Survey was developed through the Canadian Partnership Against Cancer and is based on a tool developed by Skinner [30]. The survey includes items about the participants’ demographics (e.g., role, designation, organization type, province) and degree of agreement with statements that the information was relevant and useful, increased knowledge, and that stated learning objectives were met. There are also items asking whether the participant plans to discuss the information provided at the session with colleagues or use the information to make changes in practice.

The Education Roll-Out Report was developed by the study team to collect the number of times each EPEC®-Pediatrics module was offered along with the total number of attendees at the session and whether the session was held within the hospital or in the community. Regional Teams will be asked to comment on any successes and challenges in delivering the modules, indicate any ways that the study team could provide additional supports to assist in delivery of the modules and to describe plans for delivery of sessions in the coming quarter.

#### Data analysis

**Fig. 1:**
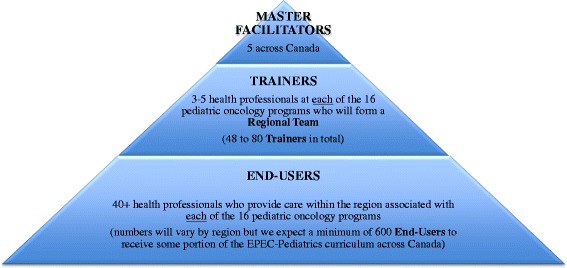
Dissemination Goals

Information obtained through the Education Roll-out Report and the KTE Surveys will be summarized descriptively. Our dissemination goal is to have a minimum of 5 Master Facilitators, 45 Trainers, and 600 End-Users from multiple professional groups across Canada (see Fig. 1) take part in at least one EPEC®-Pediatrics session. Our hypothesis is that the Trainers and End-Users will indicate that the sessions were useful, improved their knowledge related to the topic presented, and had some impact on their practice. We will compare the summarized responses to the Educational Report and KTE Surveys with these goals and hypotheses to determine if they have been achieved.

### Stream 2 - Practice change outcomes

Data collection and analysis in Stream 2 will address the impact of EPEC®-Pediatrics on practice change outcomes; namely how many of the Regional Teams initiate QI projects and achieve their practice change goals.

#### Procedure

Regional Teams at each of the 16 sites will be encouraged to complete and report on a QI project related to the overall goals of the study to improve palliative care for children with cancer. Teams may choose to use one of the TIPS Kits that are available as part of the project, or they may choose to do their own project. The EPEC®-Pediatrics curriculum currently includes one TIPS Kit on ‘symptom assessment’, with two additional kits under development related to ‘identifying goals of care’ and ‘bereavement support’. Data will be collected about the QI projects on a quarterly basis through a QI Progress Report. The report will be completed verbally on a quarterly basis with the Project Manager/PIs via telephone or Skype and data will be entered into REDCap™.

#### Data collection tool

The QI Progress Report was developed by the study team to collect information about progress made on the QI projects. Information will be collected about the practice improvement goals, strategies to achieve these goals, a summary of audit and feedback results, plans for the next quarter, as well as successes, challenges and areas for further support.

#### Data analysis

QI Progress Report Data will be summarized descriptively. Our goal is to have at least 10 of the 16 Regional Teams successfully attain the practice improvement goals set in their chosen QI project.

### Stream 3 - Quality of care

Data collection and analysis in Stream 3 will address the impact of EPEC®-Pediatrics on the quality of palliative care provided to children with cancer and their families. Quality of palliative care will be assessed using data from three sources: A) surveys about symptoms, quality of life, and care quality completed by children receiving active treatment and their parents; B) review of health records of deceased patients; and C) surveys with bereaved parents about the quality of their children’s end-of-life care. Baseline data will be collected through each of the 16 pediatric oncology programs from January to April 2015, to provide a cross-sectional view of the quality of palliative care prior to the EPEC®-Pediatrics roll-out. The same data will be collected again from September to November 2016 to determine the impact of the roll-out on care quality.

#### Sample and procedure: A) Surveys during active treatment

Parents of children with a cancer diagnosis who are receiving treatment through one of the 16 pediatric oncology programs in Canada will be invited, along with their child, to complete a survey about symptoms, quality of life, and care quality. Parents will be eligible to take part if the child is less than 19 years of age, has a cancer diagnosis, and they are able to understand and read English or French. Parents will be excluded if the child is disease-free and has not received cancer-directed therapy in the last 3 months or if the health professional involved in their care feels the family should not be approached for research participation at this time (e.g., a recent relapse, difficulty coping with child’s illness, etc.). Children aged seven years or older who meet the same criteria as the parents will be invited to complete their own survey about symptoms and quality of life.

The Parent Survey has been adapted from ‘The Survey About Caring for Children With Cancer’ (SCCC) [9, 31, 32] and the ‘Quality of Children’s End-of-Life Care Instrument’ (QCECI) [33]. The SCCC, developed by one of our co-investigators (JW), is a widely used comprehensive, self-administered survey that evaluates parents’ perceptions of the child’s illness and care quality. It was originally developed from a literature review and focus groups with parents and health professionals to identify key domains, and underwent pretesting to assess content, wording, response burden, and cognitive validity [9, 31, 32]. The QCECI was developed in a similar manner as the SCCC and tested for reliability and validity by one of the co-PIs (KW), with bereaved mothers [33]. It has also been adapted and used with parents prior to the child’s death to assess care quality. The Parent Survey will take approximately 15-30 min to complete.

The Child Survey consists of the ‘Memorial Symptom Assessment Scale’ [25, 26] and the ‘Pediatric Quality of Life Inventory’ (PedsQL™) 4.0 generic module [34] to assess symptoms and quality of life as well as questions asking how often they are asked about symptoms and quality of life by health professionals. The Child Survey will take approximately 15-20 min to complete.

Eligible children and parents will be approached during an inpatient stay or regular clinic visit by their primary nurse or other health professional actively involved in their care to see if they are interested in learning more about our study. If so, the Research Assistant (RA) will meet with the parent and child to provide further information about the study. Assent/consent will be obtained from eligible children and parents. If both parents are present, they will be asked to choose one parent to complete the survey. If the child is less than seven years old or if the child does not wish to complete a survey, the parent will still be asked to participate in the study. The RA will have hard copies of all assent/consent forms available, but the assent/consent forms will also appear in REDCap™ prior to any questions being asked. Submission of the survey signifies assent/consent. Children and parents who agree to take part will be given the option of completing the survey with the RA (i.e., the RA would access REDCap™, read the questions to the participant, and enter the responses), providing an email address where a link to the survey on REDCap™ can be sent, or taking a business card that has the survey link written on it so the participant can complete the survey on their own. Children/adolescents will be offered a certificate of completion for taking part in the study. Eligible children/parents will be approached only once during each data collection period to take part in the study; however, a child/parent who was approached during the pre-test period (Winter 2015) may be approached again during the post-test period (Fall 2016) if they still meet the eligibility criteria. Families who take part in both the pre- and post-testing period will not have their responses linked, as we are interested in cross-sectional data rather than longitudinal.

#### Sample and procedure: B) Health record reviews

Health record reviews will be conducted for all children with a cancer diagnosis who received treatment at one of the 16 pediatric oncology programs and died in the 12 months prior to the pre-test data collection (January 2014 to January 2015) and in the 6 months prior to the post-test data collection (June to November 2016).

Eligible deceased children will be identified through the Health Records Department. An on-site RA will review the health record and enter data directly into REDCap™. Data collection forms were developed based on previous health record reviews for children with cancer [9, 31, 32] and include information on diagnosis, age, location of death, involvement of a palliative care team, time spent in hospital during the last month of life, use of cancer directed treatments, and documentation of discussion of goals of care.

#### Sample and procedure: C) Bereaved parent surveys

During the pre and post-test period, bereaved parents will be invited to complete a survey about the quality of their children’s end-of-life care. Potential participants will be identified according to the following inclusion criteria: 1) Child died following a cancer diagnosis; 2) Child aged 19 years or younger; 3) Parents are able to read English or French. Parents will be excluded if: they requested to have no further contact from the hospital such as for bereavement follow-up support. Bereaved parents will not be approached about the study until at least 6 months have passed since the time of the child’s death. Therefore, in order to achieve the required sample size we will invite parents whose child died during a 2 year window that ends 6 months prior to the end of the pre-test data collection period (e.g. Invitation letters would be sent in January 2015 to bereaved parents whose child died between July 2012 and July 2014) and in an 8 month window that ends 6 months prior to the end of the post-test data collection period (e.g. Invitation letters would be sent in January 2017 to bereaved parents whose child died between December 2015 and July 2016).

The Bereaved Parent Survey was adapted from the SCCC [9, 31, 32] and QCECI [33] to mirror the survey used during active treatment, with additional items specific to the quality of end-of-life and bereavement care. Contact information (name and mailing address) of eligible parents will be obtained through Health Records. A letter about the study will be sent to eligible parents from someone who provided care to the family prior to the child’s death or who is providing bereavement follow-up. The letter will include a description of the study with instructions to access the survey online for submission via REDCap™. The letter will also provide contact information for a RA at the central study site if the parent wishes to complete the survey with the RA by telephone or to request a hard copy to complete and return by mail. When the parent accesses the survey online, the consent form will appear prior to commencing the survey questions along with a list of sources of support and information. Submission of the survey will signify consent. The invitation letter will also include a self-addressed stamped envelope to be returned to the originating site if the parent does not wish to have any further contact about the study (opt-out). If an opt-out letter has not been received, a thank you/reminder letter will be sent to all eligible parents 3-4 weeks after the initial contact.

#### Sample size

The majority of quality indicators are categorical in nature (i.e., proportion of children who die in preferred location, proportion of children/parent who report symptoms in the severe range); therefore, sample size for this component of the project was calculated based on categorical analytic techniques. Specifically, sample size for standard Chi-square analysis was conducted and increased by a factor equivalent to the assumed design effect due to the clustering within regions. Given the timeline for the project and the number of children that die from cancer across Canada in a given year (~200), we will only be able to collect data on 100 deceased children in the 6 months following project implementation. However, retrospective health record reviews and surveys can be completed for a larger number of children at baseline. A prior study examining the effect of unit level interventions across 16 pediatric units across Canada, demonstrated moderate intra-class correlations (ICCs) for knowledge outcomes, but negligible ICCs for process and clinical outcomes [35]; therefore, a small ICC of 0.01 was used to adjust the sample size estimate to account for clustering within sites. We will complete a minimum of 200 health record reviews/surveys at baseline and 100 in the post-implementation period across all 16 sites to detect a 15 % difference in outcomes with 80 % power.

#### Data analysis

Categorical data (e.g., location of care/death) and continuous data (e.g., parent reports of care quality scores) and will be summarized and descriptively reported using frequencies and proportions or means and standard deviations, respectively. To identify differences in the quality indicators between baseline and following implementation, marginal regression models (i.e. Generalized Estimating Equation models) will be used to model the outcome on time point (i.e. pre- versus post-test) and account for clustering of outcomes and practices of care within regions. All analyses will be conducted using SAS v9.3. A *p*-value of 0.05 or less will be used to indicate statistical significance.

## Discussion

Though the original EPEC™ has been available since 1999, evaluation has thus far focused on dissemination outcomes [20]; there has not been an attempt to measure the impact of EPEC™ on quality of care. However, as with most education programs for health professionals, the ultimate goal is that education will result in practice changes and better care for patients. Our study will be a significant step forward in evaluation of the impact of EPEC®-Pediatrics both on dissemination outcomes and on care quality at a national level.

Our study design and timelines will allow us to adequately measure the impact of the EPEC®-Pediatrics curriculum roll-out on self-assessed knowledge improvements of health professionals; knowledge transfer and dissemination outcomes; and practice change outcomes, as has been done in the past [20]. However, our study will be the first to examine the impact on a national level. We have chosen a pre- post-test design to evaluate our intervention in terms of its impact on quality of care. A recent systematic review indicated that a train-the-trainer model is effective in improving patient outcomes; however, the length of the intervention and timing of data collection varied widely across studies [22]. Therefore, there is little evidence available to determine an optimal length of the intervention or timing for outcome assessment that could be used to guide a stronger design, such as a cluster randomized controlled trial. It is possible that a longer intervention period and a longer follow-up period for post-test data collection may show greater improvements in patient outcomes. Additionally, though one of the strengths of EPEC®-Pediatrics is the breadth of knowledge covered in the curriculum and the flexibility that Trainers have in choosing which aspects of the curriculum to present to End-Users, this flexibility means that the roll-out may look very different across the participating sites. To address some of the limitations we will explore differences in how the curriculum has been rolled out in each site (e.g., number and type of education sessions, number of End-Users, type and success of QI projects) and whether these differences are associated with any differences in quality of palliative care across sites. This exploration may help to identify some core components of the curriculum that must be included as part of the intervention and may advance the science such that a stronger design could be used in future research to roll-out the curriculum to health professionals who care for children with life-threatening illnesses other than cancer.

Our study is powered to detect changes in care quality. However, even if no changes are seen in the course of our study, measurement of the quality of palliative care for children with cancer on a national level is an important aspect of our study and will be a significant contribution to the field of pediatric oncology and palliative care. This wealth of data can be used as the basis for future research and targeted interventions to address specific issues nationally or locally. Ultimately our goal is to enhance the care of all children who live with life-threatening illnesses; we believe this study will be a significant step forward in achieving that goal.

## Additional file

Additional file 1:
**List of Ethics Approvals.** (PDF 182 kb)

## Abbreviations

EPEC®-Pediatrics: Education in Palliative and End-of-Life Care for Pediatrics
ICC: Intra-class correlation
KT: Knowledge translation
KTE: Knowledge Transfer and Exchange
PedsQL™: Pediatric Quality of Life Inventory
PI: Principal Investigator
PPC: Pediatric palliative care
QCECI: Quality of Children’s End-of-Life Care Instrument
QI: Quality improvement
RA: Research Assistant
REDCap™: Research Electronic Data Capture
SCCC: The Survey About Caring for Children With Cancer
TIPS: Tailored Implementation of Practice Standards.
