# Supplementary material for: Protocol: Evaluating the impact of a nation-wide train-the-trainer educational initiative to enhance the quality of palliative care for children with cancer
Source: BMC Palliat Care. 2016 Jan 27;15:12. doi: 10.1186/s12904-016-0085-8 (PMC4729125; doi:10.1186/s12904-016-0085-8)
Supplement: Additional file 1: — List of Ethics Approvals. (PDF 182 kb) [file 12904_2016_85_MOESM1_ESM.pdf]

List of Ethics Approvals

Please note that 15 sites are participating in this study and have all received ethics approval from local Research Ethics Boards. The McGill University Health Centre approval is a multi-centered research approval for three participating sites: Montreal Children's Hospital, CHU de Quebec and Sainte-Justine. Please find the list of sites and supporting local approval documents included below.

	**Site**	**Research Ethics Approval**
1	The Hospital for Sick Children<br>(Toronto, ON)	Sick Kids Research Ethics Board
2	Alberta Children's Hospital<br>(Calgary, AB)	Health Research Ethics Board of Alberta (HREBA) – Cancer Committee
3	BC Children's Hospital<br>(Vancouver, BC)	University of British Columbia
4	Cancer Care Manitoba<br>(Winnipeg, MB)	University of Manitoba
5	Children's Hospital of Eastern Ontario (CHEO)<br>(Ottawa, ON)	CHEO Research Ethics Board
6	IWK Health Centre<br>(Halifax, NS)	Nova Scotia District Health Authorities Multisite Research Ethics Board (MREB)
7	Janeway Children's Health and Rehabilitation Centre<br>(St. John's, NL)	Newfoundland and Labrador Health Research Ethics Board
8	Kingston General Hospital<br>(Kingston, ON)	Queen's University Health Sciences & Affiliation teaching Hospitals Research Ethics Board (HSREB)
9	Children's Hospital, London Health Sciences Centre	Western University Health Science Research Ethics Board
10	McMaster Children's Hospital	Hamilton Integrated Research Ethics Board (HIREB)
11<br>12<br>13	Montreal Children's Hospital (Montreal QC)<br>CHU de Quebec (Charlesbourg, QC)<br>Sainte-Justine (Montreal, QC)	McGill University Health Centre<br>(Multi-centered research approval)
14	Saskatchewan Cancer Agency<br>(Regina, SK)	University of Saskatchewan
15	Stollery Children's Hospital<br>(Edmonton, AB)	University of Alberta
